# Few Differences in Metabolic Network Use Found Between *Salmonella enterica* Colonization of Plants and Typhoidal Mice

**DOI:** 10.3389/fmicb.2018.00695

**Published:** 2018-05-08

**Authors:** Grace Kwan, Brett Plagenz, Kimberly Cowles, Tippapha Pisithkul, Daniel Amador-Noguez, Jeri D. Barak

**Affiliations:** ^1^Department of Plant Pathology, University of Wisconsin-Madison, Madison, WI, United States; ^2^Department of Bacteriology, University of Wisconsin-Madison, Madison, WI, United States

**Keywords:** *Salmonella* enterica serovar Typhimurium, plant-microbe interaction, food safety, mannose metabolism, fatty acid biosynthesis

## Abstract

The human enteric pathogen *Salmonella enterica* leads a cross-kingdom lifestyle, actively colonizing and persisting on plants in between animal hosts. One of the questions that arises from this dual lifestyle is how *S. enterica* is able to adapt to such divergent hosts. Metabolic pathways required for *S. enterica* animal colonization and virulence have been previously identified, but the metabolism of this bacterium on plants is poorly understood. To determine the requirements for plant colonization by *S. enterica*, we first screened a library of metabolic mutants, previously examined in a systemic mouse typhoidal model, for competitive plant colonization fitness on alfalfa seedlings. By comparing our results to those reported in *S. enterica*-infected murine spleens, we found that the presence of individual nutrients differed between the two host niches. Yet, similar metabolic pathways contributed to *S. enterica* colonization of both plants and animals, such as the biosynthesis of amino acids, purines, and vitamins and the catabolism of glycerol and glucose. However, utilization of at least three metabolic networks differed during the bacterium's plant- and animal-associated lifestyles. Whereas both fatty acid biosynthesis and degradation contributed to *S. enterica* animal colonization, only fatty acid biosynthesis was required during plant colonization. Though serine biosynthesis was required in both hosts, *S. enterica* used different pathways within the serine metabolic network to achieve this outcome. Lastly, the metabolic network surrounding *manA* played different roles during colonization of each host. In animal models of infection, O-antigen production downstream of *manA* facilitates immune evasion. We discovered that *manA* contributed to *S. enterica* attachment, to seeds and germinated seedlings, and was essential for growth in early seedling exudates, when mannose is limited. However, only seedling attachment was linked to O-antigen production, indicating that *manA* played additional roles critical for plant colonization that were independent of surface polysaccharide production. The integrated view of *S. enterica* metabolism throughout its life cycle presented here provides insight on how metabolic versatility and adaption of known physiological pathways for alternate functions enable a zoonotic pathogen to thrive in niches spanning across multiple kingdoms of life.

## Introduction

The host ranges of most pathogens have boundaries that fall between the species and kingdom levels. However, a small group of microbes are able to transcend these boundaries to lead a cross-kingdom lifestyle, i.e., colonizing both animals and plants. For example, the nosocomial human pathogens *Enterobacter cloacae* and *Serratia marcescens* are also plant pathogens that cause bulb decay of onions and cucurbit yellow vine disease, respectively (Bishop and Davis, 1990; Hejazi and Falkiner, 1997; Sanders and Sanders, 1997; Bruton et al., 2003; Barak and Schroeder, 2012). *Burkholderia cepacia* and *Pseudomonas aeruginosa* are both responsible for pneumonia in cystic fibrosis patients as well as bacterial rots of onion (Barak and Schroeder, 2012). Dual pathogenicity, however, is not required for a cross-kingdom lifestyle. *Xylella fastidiosa* causes economically important diseases of grapes, citrus, and other plants but colonizes its insect vector without causing disease (Chatterjee et al., 2008). In order to succeed on such diverse hosts, cross-kingdom pathogens adopt strategies that range from generalist to specialist. Generalists use universal strategies to exploit animal and plant hosts, whereas specialists have dedicated mechanisms for interaction with each host (Kirzinger et al., 2011). Understanding the biology of cross-kingdom bacterial colonists on both animal and plant hosts leads to improved management of human, animal, and plant health.

This study focuses on the cross-kingdom enteric human pathogen *Salmonella enterica*, which colonizes agricultural plants as alternate hosts and uses them as a vector to humans (Barak and Schroeder, 2012). Numerous salmonellosis outbreaks linked to *S. enterica*-contaminated fresh produce have occurred in recent years; produce now ranks as the riskiest commodity for foodborne illness (Painter et al., 2013). Because the primary economic and pathological significance of *S. enterica* lies in the human-associated portion of its life cycle, the biology of this pathogen is much better understood in the context of its human and animal hosts compared to its plant hosts. However, in order to reduce foodborne illness, we need to understand the biology of *S. enterica* on plants, before the pathogen reaches humans and causes disease.

The cross-kingdom lifestyle of *S. enterica* requires that this bacterium attach to, colonize, and persist in divergent host environments. In animals, *S. enterica* colonizes the anaerobic intestinal lumen and causes gastroenteritis in humans (Wiedemann et al., 2014). *S. enterica* pathogenesis and metabolism have been best characterized in the murine host due to the tractability of mice as an experimental model. In a mouse typhoidal model, the pathogen migrates to lymphoid organs such as the spleen, invades epithelial and macrophage cells, and establishes a nutrient-limited intracellular niche called the *Salmonella*-containing vacuole (SCV) (Steeb et al., 2013; Wiedemann et al., 2014). Nutrients in the SCV are quantitatively limiting (i.e., low concentrations) rather than qualitatively limiting (i.e., few types of nutrients). In contrast to colonization of animal tissues, plant surfaces colonized by *S. enterica* are typically aerobic and sites like the rhizosphere can support high levels of bacterial replication (Kwan et al., 2015), though the nutritional drivers of this growth are still unclear. Amino acids were previously found to be limiting in alfalfa root exudates, similar to conditions encountered during colonization of the chicken intestinal lumen, but the ability of amino acid auxotrophs and transport mutants to grow in the root environment suggests that amino acid metabolism is not a determinant of *S. enterica* growth during plant association (Harvey et al., 2011; Kwan et al., 2015). A variety of sugars, organic acids, and other nutrients have also been identified on plant surfaces and in root exudates, but their contributions to plant colonization by *S. enterica* remains to be determined (El-Hamalawi and Erwin, 1986; Mercier and Lindow, 2000; Nelson, 2004; Roberts et al., 2009; Han and Micallef, 2016). The metabolic networks essential for *S. enterica* plant colonization are also largely unknown. Addressing this knowledge gap is critical to understanding how *S. enterica* succeeds on plants, a poorly-characterized part of the pathogen life cycle.

Differences in host physiologies and the host niches colonized by *S. enterica* led us to hypothesize that different metabolic networks are required for the distinct plant- and animal-associated lifestyles of *S. enterica*. In this study, we broadly examined the metabolic capabilities of *S. enterica* to identify a subset of metabolic networks that drives plant colonization by this bacterium. We used alfalfa seedlings as our model as they are the produce commodity most frequently contaminated with *S. enterica* (Callejón et al., 2015). By comparing the metabolic networks driving plant colonization by *S. enterica* to the known metabolic requirements for animal colonization, we provide a unique, integrated view of *S. enterica* metabolism throughout its life cycle.

## Materials and methods

### Bacterial strains and culture conditions

Bacterial strains and plasmids used in this study are listed in Table 1. Strains were cultured on lysogeny broth agar (LB) medium at 37°C unless otherwise indicated. When necessary, antibiotics were added at the following concentrations: ampicillin (Amp), 100 μg/ml; chloramphenicol (Cm), 40 μg/ml; kanamycin (Kan), 50 μg/ml.

**Table 1:** List of strains.

**Strain or plasmid**	**Genotype/Relevant markers**	**References**
**STRAINS**		
***S. enterica*** **sv. Typhimurium, SL1344**		
	WT; *hisG rpsL*, St^R^	Hoiseth and Stocker, 1981
	Δ*fadD*::Kan^R^ Δ*fadK*::Cm^R^, St^R^	Steeb et al., 2013
	Δ*glpFK*::Kan^R^ Δ*gldA*::Cm^R^ Δ*glpT* Δ*ugpB*, St^R^	Steeb et al., 2013
	Δ*rbsB*::Cm^R^, St^R^	Steeb et al., 2013
	Δ*galP*::Kan^R^ Δ*mglB*::Cm^R^, St^R^	Steeb et al., 2013
	Δ*nanT*::Cm^R^, St^R^	Steeb et al., 2013
	Δ*manX*::Kan^R^, St^R^	Steeb et al., 2013
	Δ*hisP*::Kan^R^ Δ*artP*::Cm^R^, St^R^	Steeb et al., 2013
	Δ*malG*::Cm^R^, St^R^	Steeb et al., 2013
	Δ*manX*::Kan^R^ Δ*nagE*::Cm^R^, St^R^	Steeb et al., 2013
	Δ*deoP*::Cm^R^, St^R^	Steeb et al., 2013
	Δ*lldP*::Kan^R^, St^R^	Steeb et al., 2013
	Δ*fucP*::Kan^R^, St^R^	Steeb et al., 2013
	Δ*eutC*::Kan^R^, St^R^	Steeb et al., 2013
	Δ*proP*::Kan^R^ Δ*proW*::Cm^R^, St^R^	Steeb et al., 2013
	Δ*ptsG*::Kan^R^ Δ*manX* Δ*mglB* Δ*galP*, St^R^	Steeb et al., 2013
	Δ*exuT*::Kan^R^, St^R^	Steeb et al., 2013
	Δ*STM4432*::Kan^R^, St^R^	Steeb et al., 2013
	Δ*idnT*::Kan^R^ Δ*gntT*::Cm^R^, St^R^	Steeb et al., 2013
	Δ*lysP*::Kan^R^ Δ*hisP*, St^R^	Steeb et al., 2013
	Δ*uhpT*::Kan^R^, St^R^	Steeb et al., 2013
	Δ*yicE*::Kan^R^ Δ*xapB* Δ*uraA* Δ*nupC* Δ*nupG*, St^R^	Steeb et al., 2013
	Δ*glgP* Δ*malP*, St^R^, Cm^R^, Kan^R^	Steeb et al., 2013
	Δ*mtlA* Δ*mtlD*, St^R^, Cm^R^, Kan^R^	Steeb et al., 2013
	Δ*glpFK*::Kan^R^ Δ*glpT* Δ*ugpB*, St^R^	Steeb et al., 2013
	Δ*glpFK*::Kan^R^, St^R^	Steeb et al., 2013
	Δ*lysA*::Kan^R^ Δ*cadA*, St^R^	Steeb et al., 2013
	Δ*sifB*::GFPweak::Kan^R^ Δ*yabJ* Δ*thiI*, St^R^	Steeb et al., 2013
	Δ*manX*::Kan^R^ Δ*manA*::Cm^R^ *sifB*@GFPs, St^R^	Steeb et al., 2013
	Δ*manA*::Cm^R^ *sifB*@GFPs, St^R^	Steeb et al., 2013
	Δ*pdxA* Δ*STM0163 sifB*@sYFP, St^R^	Steeb et al., 2013
	Δ*lysA*::Kan^R^, St^R^	Steeb et al., 2013
	Δ*ilvD*::Kan^R^, St^R^	Steeb et al., 2013
	Δ*thrC*::Kan^R^, St^R^	Steeb et al., 2013
	Δ*trpA*::Kan^R^ Δ*pheA* Δ*tyrA*, St^R^	Steeb et al., 2013
	Δ*proC*::Cm^R^, St^R^	Steeb et al., 2013
	Δ*cysE*::Kan^R^ Δ*metA*::Cm^R^ St^R^	Steeb et al., 2013
	Δ*leuB*::Cm^R^, St^R^	Steeb et al., 2013
	Δ*purH*::Cm^R^, St^R^	Steeb et al., 2013
	Δ*purA*::Kan^R^, St^R^	Steeb et al., 2013
	Δ*tsx*::Cm^R^, St^R^	Steeb et al., 2013
JDB 1332	Δ*pdxA* Δ*STM0163 sifB*@sYFP +pKTKan, St^R^	This study
JDB 1339	Δ*fabG*::CmR, St^R^	This study
JDB 1343	Δ*fabG*::CmR + pEVS141(Kan^R^), St^R^	This study
JDB 1344	Δ*fabG*::CmR + pEVS141Δ*gfp*::*fabHDG*(Kan^R^), St^R^	This study
JDB 1336	Δ*gmd*::Kan^R^, St^R^	This study
JDB 1337	Δ*manA*::Cm^R^, St^R^	This study
JDB 1340	Δ*manA*::Cm^R^ + pEVS141(Kan^R^), St^R^	This study
JDB 1341	Δ*manA*::Cm^R^ + pEVS141Δ*gfp*::*manA*(Kan^R^), St^R^	This study
JDB 1399	Δ*serA*::Cm^R^, St^R^	This study
JDB 1400	Δ*serA*::Cm^R^ + pEVS141Δ*gfp*::*serA*(Kan^R^), St^R^	This study
JDB 1342	WT + pEVS141(Kan^R^)	This study
***S. enterica*** **sv. Typhimurium, 14028S**		
	WT	ATCC
	Δ*asnA*::Kan Δ*asnB*::Cm	Kwan et al., 2015
	Δ*glyA*::*Tn*10d	Kwan et al., 2015
	Δ*lysA*::Kan	Kwan et al., 2015
	Δ*metC*::Kan	Kwan et al., 2015
	Δ*pheA*::Kan	Kwan et al., 2015
	*proC693*::*Mu*dA	Kwan et al., 2015
	Δ*trpB*::Kan	Hao et al., 2012
	Δ*tyrA*::Kan	Kwan et al., 2015
	Δ*rfbP*::Kan^R^	Santiviago et al., 2009
JDB 1358	Δ*gmd*::Kan^R^	This study
**PLASMIDS**		
pKTKan	*gfp*, Kan^R^	Miller et al., 2000
pEVS141	*gfp*, Kan^R^	Dunn et al., 2006

### Mutant construction

Gene deletion mutants were generated in *S. enterica* KHLT2 with the λ-Red recombination method (Datsenko and Wanner, 2000) and transduced into *S. enterica* 14028S or *S. enterica* SL1344 by bacteriophage P22. A *S. enterica* 14028S Δ*rfbP*::Kan mutant was obtained from a deletion library generated by the same method (Santiviago et al., 2009). Due to lack of O-antigen production, this mutant could not be transduced into the SL1344 background. All mutants were confirmed by PCR, either with two flanking verification (“veri”) primers or one flanking verification primer and an antibiotic-cassette specific primer (c1 or k2) (Datsenko and Wanner, 2000). Mutagenesis and verification primers are listed in Table 2. Double mutants were produced by transduction into a mutant background.

**Table 2:** Primers used in this study.

**Primer name**	**Primer sequence (5′3′)**
Λ**-RED DELETION/VERIFICATION**	
fabG-RS-F	GGCGCTGTCTGCGGCACTTACGCAATAAAAGAGGAAAACCG
	TGTAGGCTGGAGCTGCTTC
fabG-RS-R	GGCCTTTCGCCCTAATAACGCAAATATTTTTCAATCGTGACA
	TATGAATATCCTCCTTAG
fabG-RS-veriF2	GAAACCAGCGGCAGATAAGC
manA-RS-F	ATACCTCCCATTGATCTCCACATTGAAACAGGGCTTGATAGT
	GTAGGCTGGAGCTGCTTC
manA-RS-R	TATAAGCTTAGCAAGAGTTGTTAAAAAATTCAGTACGTTGCA
	TATGAATATCCTCCTTAG
manA-RS-veriF2	CCTGTCCAGACGATGCCAAG
gmd-RS-F	AGAAAGTTACTCCCTAACGGGACTATTTGAGGAAATGAAAGT
	GTAGGCTGGAGCTGCTTC
gmd-RS-R	CCGCGATGGCCCGCCACAAAAATTCGTTGCTTATTCATTCCA
	TATGAATATCCTCCTTAG
gmd-veriR	CGTGACGATAGGTCATGGCA
STM2084-veriR2	CGGCTTTTCATCGCCTGTTG
rfbP-veriF	CTTTCGATGTTGAGCGCGAG
rfbP-veriR	GCACCTGAGTTACGCTGCTA
serAredFor	AAAAGACAGGATCGGGGAAATGGCAAAGGTATCGCTGGAGGTGTAGGCTGGAGCTGCTTC
serAredRev	GCAGGTCATCTCCTGCCCATTTAGCGGAAATTAGTACAGCCATATGAATATC CTC CTTAG
serAconfirmFor	TTCCTTCACGACGCTGGC
serAconfirmRev	TGTTGAGGCAGGGAAACC
C1	TTATACGCAAGGCGACAAGG
K2	CGGTGCCCTGAATGAACTGC
**COMPLEMENTATION**	
fabH-AvrII-F	NNNCCTAGGTCGTGGTGGCTGCTGTTATT
fabG-BamHI-R	NNNGGATCCTCTTCCTGCTTAACGCCCAG
manA-AvrII-F	NNNCCTAGGTGTCCAGACGATGCCAAGTG
manA-BamHI-R	NNNGGATCCCGCGGGACGTAGCATAAAAG
serAForAvr	NNNCCTAGGAGGGTTGTTCGCCAACCG
serARevBam	NNNGGATCCACCAGAGAAAGGATGGGC

### Complementation

The Δ*manA*::Cm, Δ*fabG*::Cm, and Δ*serA*::Cm strains were genetically complemented by cloning of the *manA, fabHDG*, or *serA* genes into pEVS141. The complementation plasmids were transformed into the respective mutant strains. Transformants were selected on LB+Kan and confirmed by PCR of the cloned gene(s) from extracted plasmid DNA. The same primers were used for cloning and PCR confirmation (Table 2). For chemical complementation, 0.2 mM mannose was provided to Δ*manA*::Cm; 0.1 mM cysteine was added as a supplement for Δ*cysE* Δ*metA*.

### Preparation of inoculum

For all experiments except mutant analysis of *serA*, overnight bacterial cultures were suspended in sterile deionized water and normalized to ~10^8^ CFU/ml by absorbance at OD_600_ using a spectrophotometer. This suspension was diluted as needed. For experiments involving Δ*serA*::Cm, the WT, mutant, and complement bacterial suspensions were made from overnight cultures grow in liquid lysogeny broth rather than LB agar. Inoculum populations were verified by dilution plating on LB.

### Plant assays

Surface sanitized alfalfa seeds (International Specialty Supply) were prepared as described by Cowles et al. (2016).

For alfalfa attachment assays, 10 seeds or 10 3-day-old seedlings were placed in sterile 50 ml conical tubes and co-inoculated with 20 ml of a 10^3^ CFU/ml bacterial suspension of wild-type (WT) and mutant at a 1:1 (±0.1) ratio. For experiments using Δ*manA*::Cm, the WT, mutant, and complement bacterial suspensions were made from overnight cultures on M9 minimal agar media amended with 0.4% glucose and 0.2 mM histidine because LB likely contains low concentrations of mannose (our own unpublished results). Histidine was required because *S. enterica* SL1344 is a histidine auxotroph. For chemical complementation, mannose was added to this overnight culture because the surface polymers produced downstream of *manA* (see Discussion) that are known to mediate attachment must be pre-formed in culture; *S. enterica* does not grow during the first 4 h following inoculation into germinating seedling cultures–the time period used for this attachment assay (Becker et al., 2006; Kwan et al., 2015). Tubes were incubated for 4 h at room temperature with gentle shaking. Then, the water was decanted and seeds or seedlings were rinsed twice in 20 ml sterile water by vortexing on high for 30 s to remove loosely attached bacterial cells. Individual seeds or seedlings were homogenized in 300 ul sterile water and plated on LB and LB+Kan or LB+Cm. Plates were incubated at 37°C overnight, and populations were enumerated. The experiment was repeated at least three times.

For alfalfa colonization assays, 0.3 g surface sanitized alfalfa seeds were placed in plastic petri dishes and irrigated with 20 ml of a bacterial suspension containing 10^5^ CFU/ml. For co-inoculation (competition) assays, the bacterial suspension contained a 1:1 (±0.1) ratio of two bacterial strains. After a 1 h incubation at room temperature with constant gentle agitation, the irrigation water was replaced with fresh sterile water. Inoculated seeds were then incubated at 24°C on a rotating shaker set at ~40 rpm and under constant light. Every 24 h, samples were removed, and the irrigation water was replaced. Seedlings with attached seed coats were sampled at 24 h post-inoculation, and those without seed coats were selected at 48 and 72 h. At each time point, three seedlings were placed in a 50 ml conical tube containing 20 ml sterile water and vortexed on high for 30 s. Then, individual seedlings were homogenized in 500 μl sterile water and dilution plated on LB, LB+Kan, and/or LB+Cm. Plates were incubated at 37°C overnight, and then populations were enumerated. There were three replicates per treatment and the experiment was repeated at least three times. Because it was necessary to distinguish between WT and mutant cells in our competition assays (detailed below), pKT-Kan was transformed into the unmarked Δ*pdxA* ΔSTM0163 strain as a selection marker. No growth difference was observed between the marked and unmarked strains in LB (data not shown). For chemical complementation, the appropriate metabolite was added into the irrigation water at each water change.

For growth assays in the exudates of germinating plant seedlings, experiments were set up as described for the colonization assays above except that the inoculum concentration was 10^3^ CFU/ml and the irrigation water was never changed. Growth was examined in the exudates of three different plants: alfalfa, broccoli, and lettuce. Broccoli seeds (Johnny Seeds; Winslow, ME) were surface sanitized as described above for alfalfa seeds. Lettuce seeds (cultivar Green Towers; GeneFresh Technologies; Salinas, CA) were not sanitized because bleach sanitized seeds failed to germinate. Approximately 130 alfalfa (=0.3 g), lettuce (=0.125 g), or broccoli seeds (counted by hand) were placed in each petri plate. Planktonic bacterial cell populations were determined every 24 h, for 72 h, by removing 100 ul of the irrigation water and plating serial dilutions on LB. Plates were incubated at 37°C overnight and then populations were enumerated. No bacterial growth was observed for control samples that were not inoculated (data not shown). There were three replicates per treatment, and the experiment was repeated at least three times.

To determine Δ*manA*::Cm growth in more mature plant exudates, the experiment was conducted identically to the typical growth assay (above) except that alfalfa seeds were germinated in sterile water for 24 h prior to bacterial inoculation. This method contrasts with the normal experimental setup where bacteria were inoculated into sterile water containing yet ungerminated seeds.

### Seedling toxicity assays

Alfalfa seeds (0.3 g) were germinated in 20 ml of a 0.1 mM cysteine solution and incubated for 3 days at 24°C on a rotating shaking set at ~40 rpm and under constant light. Seeds germinated in sterile water served as the control. The irrigation water was removed and replaced with sterile water or 0.1 mM cysteine daily. The length of five 3-day-old seedlings was determined using a ruler. The experiment was repeated three times.

### Metabolomics

Metabolites in alfalfa seedling exudates were identified by liquid chromatography-mass spectrometry (LC-MS) as described by Kwan et al. (2015). Briefly, 0.3 g surface sanitized alfalfa seeds were germinated in 20 ml of a *S. enterica* 14028S bacterial suspension (10^3^ CFU/ml) or mock-inoculated with sterile water. At 0, 8, 24, 48, and 72 h, the irrigation water was removed, passed through a 0.22 μM PES membrane filter, and analyzed by LC-MS to identify metabolites present in alfalfa seedling exudates. At least two biological replicates, each produced by pooling three technical replicates collected concurrently, were analyzed for each treatment at each time point.

### Statistical analyses

Statistical analyses were performed using R software (version 2.14.1; R Development Core Team, R Foundation for Statistical Computing, Vienna, Austria [http://www.R-project.org]). Bacterial population counts were log_10_-transformed prior to analysis except in seed and seedling attachment assays where counts remained untransformed. The mean population size (single inoculation) or percent total population (co-inoculation) were calculated at each time point for each experiment and then averaged across experiments. Biological replicates within an experiment were considered subsamples. For plant attachment assays, the percent of the total population comprised of the mutant at each time point was compared against the percentage of the mutant in the inoculum in paired, two-tailed *t*-tests assuming unequal variance. Differences among treatments at each time point in singly inoculated growth (alfalfa) and seedling toxicity were also evaluated by paired, two-tailed *t*-tests assuming unequal variance. Differences in growth between amino acid auxotrophs and WT in lettuce and broccoli seedling exudates were calculated. These differences were rank transformed (Conover, 2012), then compared to an expected value μ, 0, using one-sample *t*-tests. For plant co-colonization and co-growth assays, the average percent total population for each mutant was compared against the expected value of μ, 50, in one-sample *t*-tests. For plant complementation assays, differences among treatments at each time point were analyzed using Tukey's honestly significant difference (HSD) test (based on two-way analysis of variance [ANOVA] with strain and experiment as factors). Significance was set at a *p* < 0.05 for all aforementioned tests.

The ratios of nutrient concentrations in the presence and absence of *S. enterica* were compared against an expected μ value of 1 using one-sample *t*-tests (*p* < 0.1). A ratio of 1 indicates that *S. enterica* had no effect on the concentration of a particular nutrient in alfalfa exudates; a ratio less than 1 indicates nutrient depletion in the presence of *S. enterica*.

## Results

### Metabolic network requirements for *S. enterica* plant colonization

**Figure 1:**
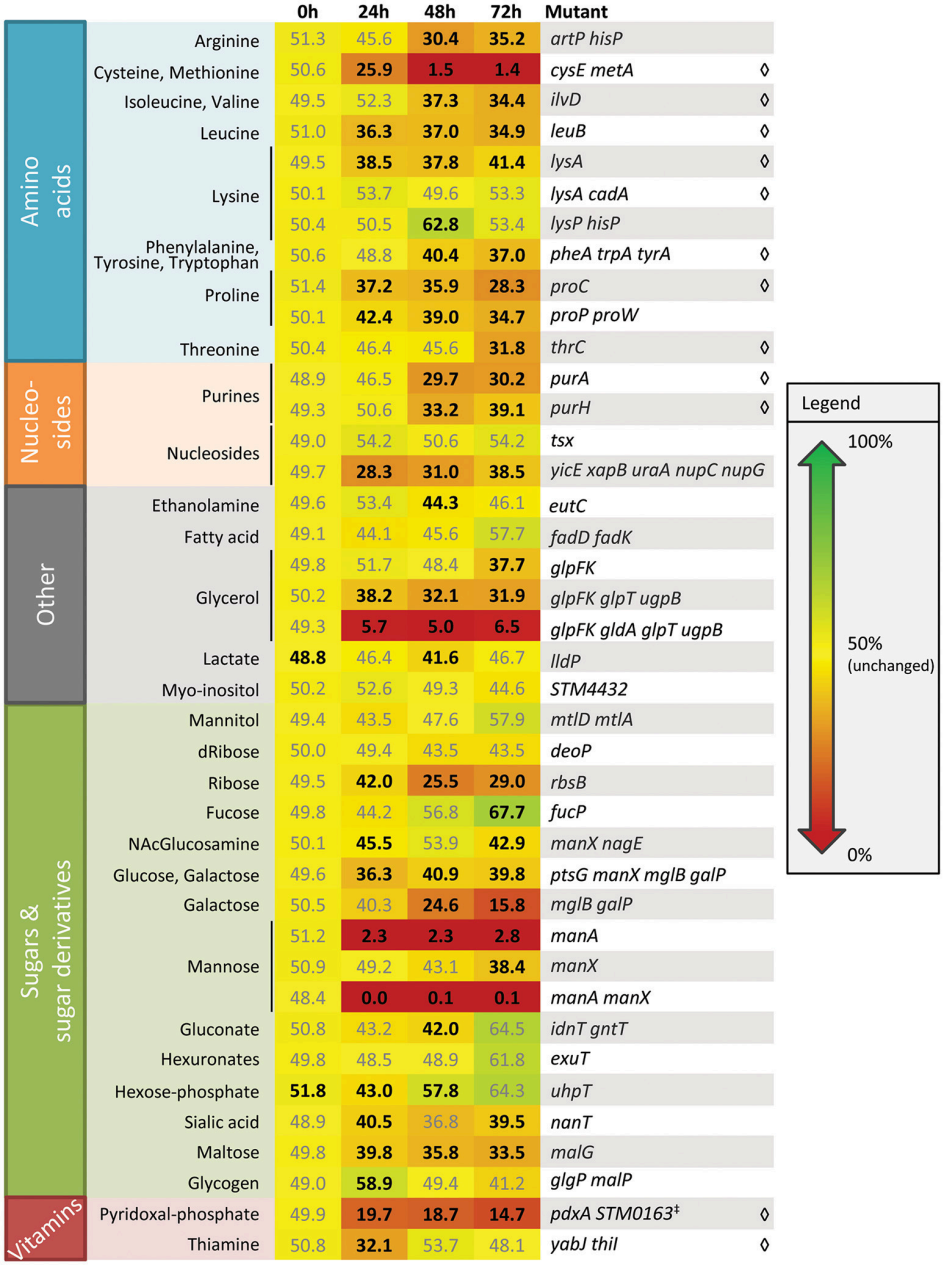
Alfalfa seedling colonization fitness of *Salmonella* SL1344 metabolic mutants. Values indicate the percent of the total population comprised of mutant, following a 1:1 co-inoculation of WT and mutant. Cells containing these values are color coded as a heat map from 0 (red) to 100% (green) with yellow as 50%, i.e., equal to WT, for easy visualization of the data. Auxotrophic mutants are indicated by ♢; all other mutants lack genes for utilization of specific nutrients. The data shown are the means of three independent experiments with three replicates each. Bold values indicate a statistically significant difference from 50% (*t*-test, *p* < 0.05).

To identify metabolic networks that contribute to plant colonization by *S. enterica*, we screened a library of *S. enterica* SL1344 mutants for colonization fitness on alfalfa seedlings (Figure 1). This set of mutants had previously been tested for spleen colonization in a mouse typhoid fever model (Steeb et al., 2013). Mutants defective in the biosynthesis of amino acids, vitamins, and purines were outcompeted by the isogenic WT, suggesting that these nutrients are limiting in or on seedling surfaces. Amino acid auxotrophs were also reduced in growth in alfalfa (Kwan et al., 2015), broccoli, and lettuce seedling exudates, indicating that amino acid limitation is common in the plant environment (Table S1). A *cysE metA* mutant, unable to biosynthesize the sulfur-containing amino acids cysteine and methionine, was severely compromised in its competitive colonization fitness vs. the WT, especially at 48 and 72 h. This fitness defect was partially complemented by addition of 0.1 mM cysteine but alfalfa seedlings were severely stunted (Figure S1). Seedlings germinated in the presence of cysteine were ~50% shorter than their counterparts irrigated with water only, indicating that this amino acid is toxic to alfalfa development. Biosynthesis of many compounds is required for *S. enterica* colonization of both plant and animal hosts.

In addition to biosynthesis, nutrient transport also contributes to *S. enterica* plant colonization (Figure 1). A *glpFK gldA glpT ugpB* mutant, unable to transport or utilize glycerol and glycerol-3-phosphate, had a severe competitive disadvantage vs. the WT, suggesting that glycerol and glycerol-3-phosphate are major nutrient sources that drive *S. enterica* seedling colonization. Based on the mutant data, glucose, galactose, ribose, maltose, sialic acid, nucleosides, arginine, and proline are also likely present in the seedling environment and used to support bacterial colonization of seedlings. The temporal releases of nutrients in alfalfa exudates that can serve as sole carbon or nitrogen sources for *S. enterica*, as inferred from the mutant data and as directly measured by LC-MS (Tables S2–S4) are detailed in Table 3. Utilization of a variety of plant-derived nutrients, likely simultaneously, contributes to bacterial fitness during plant colonization.

**Table 3:** Temporal utilization of nutrients by *S. enterica* in germinating alfalfa exudates that serve as sole C- or N-sources* or vitamins.

**Nutrient type**	**Nutrient**				**24 h**	**48 h**	**72 h**
Amino acids	Aspartate	‡			X	X	X
	Glutamine	‡	§		X	X	X
	Glycine		§		X	X	X
	Proline	‡	§		X	X	X
	Serine	‡	§		X	X	X
	Alanine	‡			-	X	X
	Arginine	‡	§		-	X	X
	Asparagine	‡			-	X	X
Nucleosides	Adenosine	‡			-	X	X
	Guanosine	‡			-	X	X
	Uridine	‡			-	X	X
	Inosine	‡			-	X	X
Organic acids	Fumarate	‡			-	X	X
	Glycerate	‡			-	X	X
	Malate	‡			-	X	X
	Succinate	‡			-	X	X
Polyols	Glycerol-3-P		§		X	X	X
	Glycerol		§		–	-	X
Sugars	Maltose		§		X	X	X
	Ribose		§		X	X	X
	Glucose		§		X	-	-
	Galactose		§		-	X	X
Vitamins	Biotin	‡			-	X	X
	Panthothenate	‡			-	X	X
	Pyridoxal-P		§		X	X	X
	Thiamine	‡	§		X	X	X

*Compounds that can be used as sole carbon and nitrogen sources by S. enterica were identified by Gutnick et al. (1969). ‡, Nutrient utilization directly measured by LC-MS, as reported in Kwan et al. (2015) and this work. §, Nutrient utilization inferred from mutant data. Glutamine utilization inferred from Kwan et al. (2015). X, present and utilized by S. enterica as a nutrient. -, not present or no significant utilization by S. enterica as a nutrient. All nutrients measured by LC-MS were detected in plant exudates at all time points indicated by “-.” Inferences made from mutant phenotypes were unable to distinguish between nutrient absence vs. lack of utilization by S. enterica.

### Fatty acid biosynthesis, but not catabolism, is required for plant colonization by *S. enterica*

**Figure 2:**
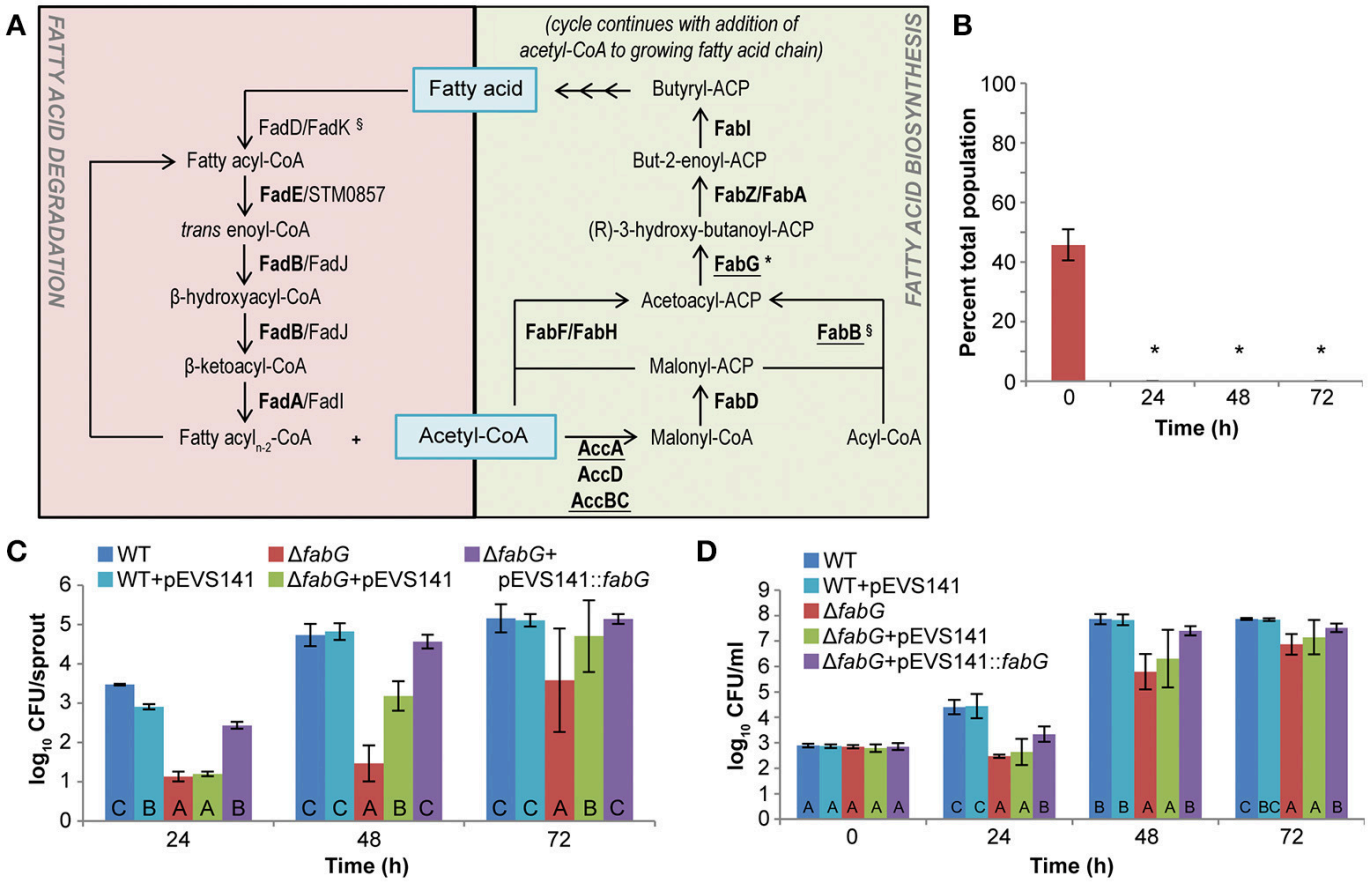
The fatty acids metabolic network is required for *S. enterica* colonization of plants and animals. **(A)** Fatty acid degradation contributed to *S. enterica* colonization of animals (Steeb et al., 2013) whereas fatty acid biosynthesis was required for colonization of both animals and plants. Fatty acid network proteins were detected in proteomic screens of *S. enterica* during colonization of mice (bolded, Steeb et al., 2013) and growth in association with alfalfa seedlings (underlined, Kwan et al., 2015). ^§^, significant colonization defect of mutant in mice (Barat et al., 2012; Steeb et al., 2013). ^*^, significant colonization defect of mutant on alfalfa seedlings. **(B)** *S. enterica* SL1344 Δ*fabG* was non-competitive in alfalfa seedling colonization. Bars show the mean percent of the total population comprised of Δ*fabG* following a 1:1 co-inoculation with the isogenic WT. ^*^, statistically different from 50% (Student's *t*-test, *n* = 3, *p* < 0.05). **(C,D)** Δ*fabG* was defective in alfalfa seedling colonization **(C)** and growth in seedling exudates **(D)**. These defects were complemented by addition of a plasmid-borne copy of *fabG* on pEVS141. Bars show the average population of *S. enterica* present on alfalfa sprouts **(C)** or the irrigation water containing seedling exudates **(D)**. Letters indicate statistically different groups within each time point (Tukey's HSD, *n* = 45, *p* < 0.05). For all experiments, data shown are the means of 3 independent experiments with 3 replicates each. Error bars indicate the standard deviation.

Proteomic data indicate that the *S. enterica* fatty acid metabolic network is active during mouse spleen colonization and alfalfa seedling-associated growth (Figure 2A; Steeb et al., 2013; Kwan et al., 2015). Both degradation and biosynthesis of fatty acids are active in the animal model of infection and mutants defective in these pathways are less fit (Becker et al., 2006; Steeb et al., 2013). In contrast, fatty acid degradation appears to be inactive or dispensable for plant colonization. No proteins involved in β-oxidation of fatty acids were detected in a shotgun proteomic survey of the *S. enterica* proteome during growth in alfalfa seedling exudates (Kwan et al., 2015), and a *fadD fadK* mutant, unable to transport or activate fatty acids for β-oxidation, had no loss of fitness during competitive colonization of alfalfa seedlings (Figure 1). However, five proteins involved in fatty acid biosynthesis (AccA, AccB, AccC, AccD, and FabB) were identified in the *S. enterica* proteome during seedling-associated growth (Kwan et al., 2015), suggesting that this metabolic pathway was both active and required by *S. enterica* in this environment. To test this hypothesis, we examined a *fabG* mutant, defective in fatty acid biosynthesis, and found that it was non-competitive against the WT for seedling colonization (Figure 2B). The *fabG* mutant never exceeded 0.06% of the total bacterial population and frequently no *fabG* colonies were recovered. This mutant also exhibited a severe colonization defect even in the absence of competition from WT (Figure 2C). To better understand the nature of the defect, we examined the *fabG* mutant in each of the two distinct phases of plant colonization, attachment to plant surfaces followed by microbial growth. The *fabG* mutant was as competitive as WT for attachment to alfalfa seeds and 3-day-old seedlings (data not shown) but showed a growth defect in germinating alfalfa seedling exudates (Figure 2D). Both the growth and colonization defects of the *fabG* mutant were complemented by a plasmid-borne WT copy of *fabG* (Figures 2C,D). Taken together, fatty acid biosynthesis is not just a fitness factor but a determinant of growth during early seedling colonization by *S. enterica*.

### Serine biosynthesis is required for plant colonization by *S. enterica*

**Figure 3:**
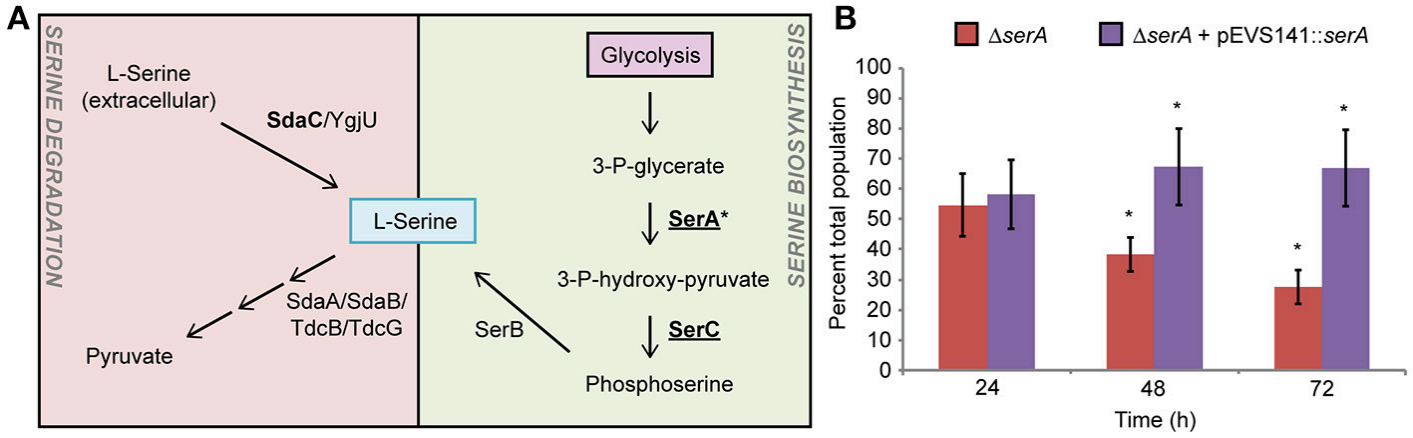
Serine biosynthesis is required for *S. enterica* plant colonization. **(A)** Serine network proteins were detected in proteomic screens of *S. enterica* during colonization of mice (bolded, Steeb et al., 2013) and growth in association with alfalfa seedlings (underlined, Kwan et al., 2015). *S. enterica* SL1344 Δ*serA* was reduced in competitive fitness in alfalfa seedling colonization **(B)**. Bars show the mean percent of the total population comprised of Δ*serA* following a 1:1 co-inoculation with the isogenic WT. ^*^, statistically different from 50% (Student's *t*-test, *n* = 3, *p* < 0.05).

During alfalfa seedling-associated *S. enterica* growth and animal infection, serine metabolic network activity was detected (Figure 3A; Steeb et al., 2013; Kwan et al., 2015) and yet, serine biosynthesis is dispensable for mouse infection (Jelsbak et al., 2014). A *serA* mutant was poorly competitive for alfalfa seedling colonization at 48 and 72 hpi (Figure 3B) and grew poorly in root exudates (data not shown). The fitness of the *serA* mutant was fully restored by genetic complementation by a plasmid carrying a WT copy of *serA*. These data suggest that serine biosynthesis occurs during both plant and animal colonization but is only essential during seedling colonization.

### *manA* Is required for *S. enterica* plant colonization

**Figure 4:**
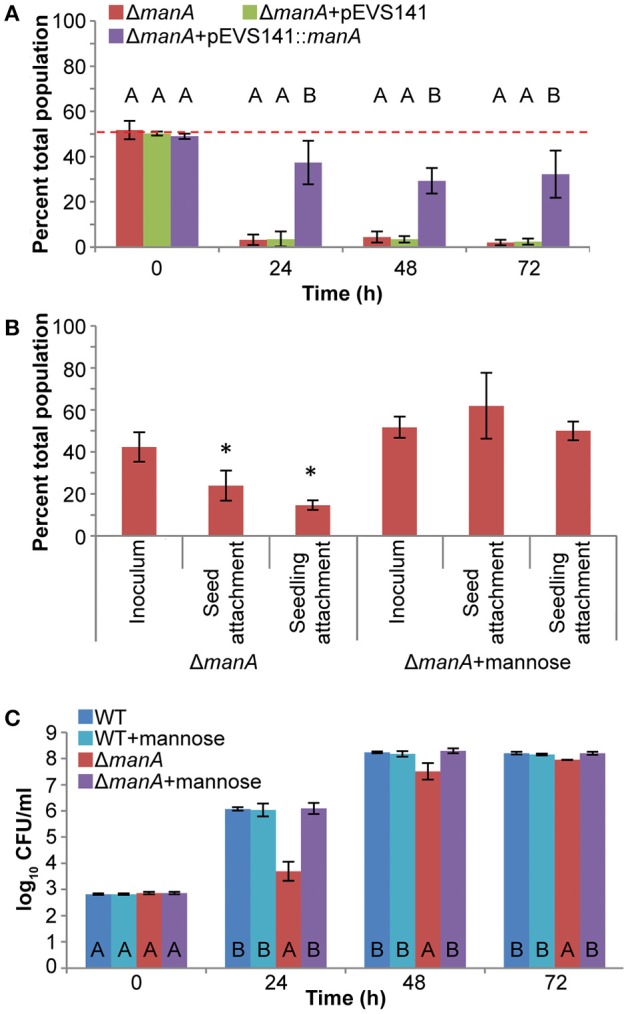
Mannose is required for *S. enterica* plant colonization. **(A)** SL1344 Δ*manA*::Cm had a severe competitive colonization defect on alfalfa seedlings. Bars show the mean percent of the total population comprised of Δ*manA* following a 1:1 co-inoculation with the isogenic WT. ^*^, statistically different from 50% (One-sample *t*-test, *n* = 3, *p* < 0.05). Letters above bars, statistically different groups within each time point (Tukey's HSD, *n* = 9, *p* < 0.05). **(B)** Mannose contributed to *S. enterica* attachment to alfalfa seeds and 3 day-old seedlings. The attachment defect of Δ*manA*::Cm was fully complemented by addition of 0.2 mM mannose. Bars show the mean percent of the total population comprised of Δ*manA* following a co-inoculation with the isogenic WT. ^*^, statistically different from the inoculum (Paired *t*-test, *n* = 6, *p* < 0.05). **(C)** Mannose contributes to *S. enterica* growth in alfalfa seedling exudates *in vivo*. The growth defect of Δ*manA*::Cm was fully complemented by addition of 0.2 mM mannose. Letters at the bottom of the bars, statistically different groups within each time point (Tukey's HSD, *n* = 36, *p* < 0.05). For all experiments, data shown are the means of 3 independent experiments with 3 replicates each. Error bars indicate the standard deviation.

Proteomic data indicate that ManA is active during mouse spleen colonization and a *manA* mutant is less fit in infected mouse tissues (Steeb et al., 2013). A *manA* mutant was also non-competitive for alfalfa seedling colonization (Figure 1). ManA catalyzes the reversible isomerization of mannose-6-phosphate into fructose-6-phosphate. This defect was partially complemented by a plasmid carrying a WT copy of *manA* (Figure 4A). The fitness of the *manA* mutant was fully restored by addition of 0.2 mM mannose, but not 0.2 mM fructose, suggesting that the directionality of the reaction catalyzed by *manA in planta* is toward production of mannose-6-phosphate (data not shown). Mannose-6-phosphate is a precursor for the biosynthesis of the surface polymers colanic acid and O-antigen. The competitive colonization defect of the *manA* mutant stemmed from defects in both attachment, to alfalfa seeds and seedlings, and growth (Figures 4B,C). These defects were fully complemented by the addition of 0.2 mM mannose to the overnight culture (attachment) or the irrigation water (growth). The growth pattern of the *manA* mutant suggested that growth was delayed, but the growth rate was not impaired beyond the first 24 h. During the first 24 h, WT populations increased ~3 log_10_ (1000-fold) whereas the *manA* mutant growth was severely restricted to <1 log_10_ (<10-fold; Figure 4C). The *manA* mutant resumed growth between 24–48 h, increasing 3.8 log_10_, resembling WT replication during the 0–24 h period. Taken together, these data suggest that mannose may be a required metabolite that is absent from early seedling exudates. Alternately, *manA* may be required for plant host interactions.

### O-antigen contributes to distinct steps in plant colonization

**Figure 5:**
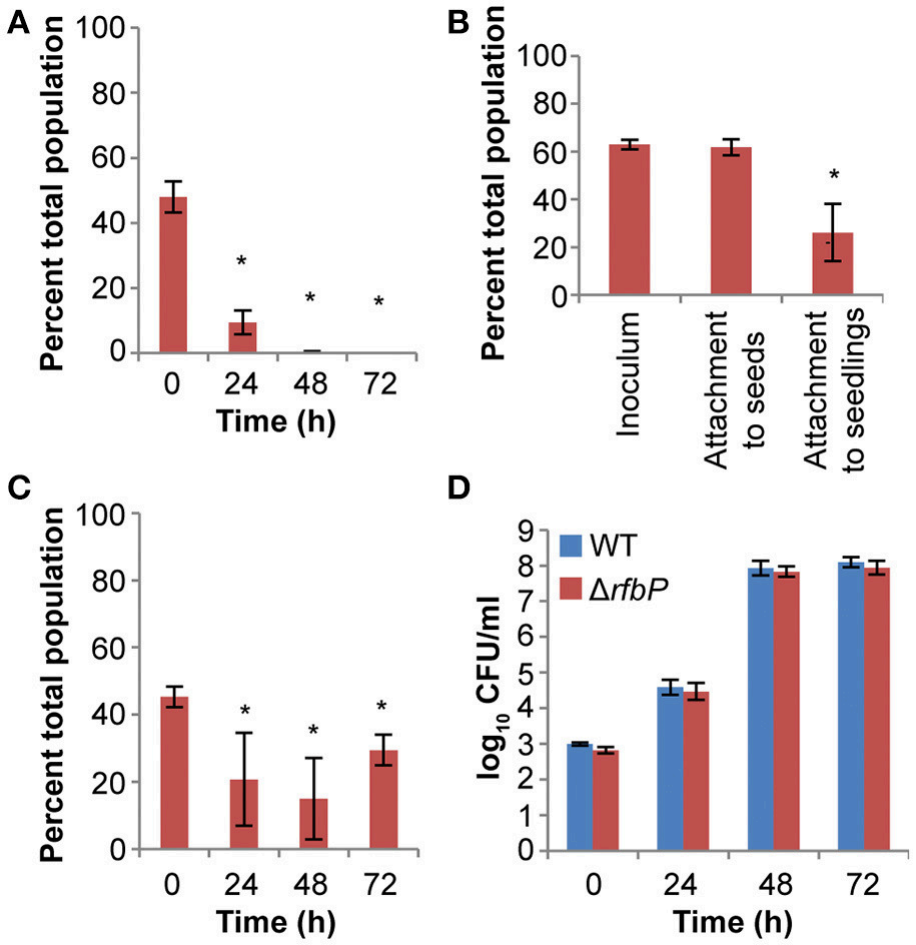
O-antigen contributes to *S. enterica* plant colonization. **(A)** 14028S Δ*rfbP*, an O-antigen mutant, was less competitive than the isogenic WT in alfalfa seedling colonization. ^*^, statistically different from 50% (Student's *t*-test, *n* = 3, *p* < 0.05). **(B)** O-antigen contributed to attachment to 3 d-old seedlings but not seeds. ^*^, statistically different from inoculum (Paired *t*-test, *n* = 6, *p* < 0.05). **(C,D)** Δ*rfbP* had a subtle growth defect in alfalfa seedling exudates that is revealed only in competition with the isogenic WT. **(C)** ^*^, statistically different from 50% (Student's *t*-test, *n* = 3, *p* < 0.05). **(D)** ^*^, statistically different from WT (Paired *t*-test, *n* = 6, *p* < 0.05). WT and mutant were inoculated at a 1:1 ratio for all competition experiments **(A,B,C)**. All data shown represent the means of 3 independent experiments with 3 replicates each. Error bars indicate the standard deviation.

We hypothesized that the colonization defects of the *manA* mutant were due to defects in the production of extracellular polysaccharides from the mannose metabolic pathway, either O-antigen or colanic acid. We tested this hypothesis by examining the competitive colonization of *gmd* and *rfbP* mutants against the WT. A *gmd* mutant, unable to produce the GDP-fucose, is defective in colanic acid biosynthesis and a *rfbP* mutant fails to catalyze the first step in O-antigen assembly on the bacterial cell membrane. The *gmd* mutant was as competitive as the WT for seedling colonization and exhibited no attachment or growth defects (data not shown). The *rfbP* mutant was less fit than the WT in a competitive colonization assay (Figure 5A). This colonization defect was due to deficiencies in attachment and competitive growth (Figures 5B,C, respectively). However, no growth defect was observed when the *rfbP* mutant was singly inoculated into plant cultures (Figure 5D). Our data indicate that O-antigen, but not colanic acid, contributed to distinct steps of *S. enterica* seedling colonization.

## Discussion

It is widely accepted that *S. enterica* adopts a cross-kingdom lifestyle, actively colonizing and persisting on plants in between infection of animal or human hosts (Barak and Schroeder, 2012). We hypothesized that this human pathogen would employ distinct metabolic strategies for colonization of cross-kingdom hosts due to the inherent differences in plant and animal host physiology as well as the nature of the extracellular (plant) vs. intracellular (animal) niches. A recent mutant screen of metabolic mutant colonization of murine spleens gave us the opportunity to test our hypothesis. Direct comparison of the same set of *S. enterica* mutants in both environments is a powerful approach that avoids identification of artificial differences that may arise from comparisons based on disparate data sets. Whereas the SCV inside of animal cells contains high levels of reactive oxygen and nitrous oxide species and few sugars but sufficient amino acids, plant root exudates are aerobic and sugar-rich, but amino acid deficient. We were thus surprised to find that *S. enterica* generally depends on the same metabolic networks to exploit both plant and animal hosts.

Biosynthesis of essential cellular metabolites is a key metabolic function shared between the plant- and animal-associated lifestyles of *S. enterica*. However, the specific nutritional constraints on *S. enterica* growth differed between plant and animal hosts. Though amino acids were present in both host environments, as inferred from impaired colonization by mutants and measured depletion of these metabolites, amino acids were much more limiting during growth in plant seed exudates (alfalfa, lettuce, broccoli; Kwan et al., 2015 and this study) and tomato fruit (Han and Micallef, 2016) than in animal models of infection. Of the 13 amino acids which have been examined in murine models, only asparagine and proline biosynthesis were required for full fitness (Steeb et al., 2013; Jelsbak et al., 2014). In contrast, *de novo* biosynthesis of at least 8 different essential amino acids (out of 18 examined) were required in each plant environment for full *S. enterica* fitness (Kwan et al., 2015; de Moraes et al., 2017, and this study), though the exact amino acids varied by plant. Notably, methionine and tryptophan biosynthesis emerged as common requisites for the plant-associated lifestyle of *S. enterica* that are dispensable during animal infection. We recognize that levels of different amino acids will vary and depend on factors such as plant growth conditions, plant genotype, plant growth stage, as well as plant organ; yet, our study did examine root exudates from plants from three distinct orders while murine models are restricted to a single host (mouse) that carry a dysfunctional *Slc1 1a1* allele which makes them highly susceptible to systemic *Salmonella* infections. Collectively, the data presented herein and in the current literature indicate that the host-associated lifestyles of *S. enterica*, whether on plants or animals, involve exploitation of host nutrients as well as *de novo* biosynthesis of metabolites insufficiently available from the host.

The much greater variety of amino acids that must by produced endogenously during plant-associated growth compared to animal niches reflects the general low availability of these nutrients from plant hosts as well as the high bacterial titers (≥10^7^ CFU/ml) in plant environments. For example, the average concentration of every amino acid except threonine in alfalfa seedling exudates was less than 70 uM, which is 3–185 times lower than reported in murine spleen tissue (Xiao et al., 2016). Plants may tightly control production and release of certain nutrients, e.g., cysteine, due to their toxicity (Figure S1). Thus, even dedicated plant colonists such as *P. fluorescens* and *P. tolaasii* must synthesize these metabolites to succeed in the plant environment (Simons et al., 1997; Chung et al., 2014). Because amino acid restriction is common in the plant environment, amino acid prototrophy appears as a universal plant colonization trait for a variety of plant-associated bacteria.

Utilization of multiple, diverse nutrients appears to be a general metabolic colonization strategy of *S. enterica*. During typhoid fever in mice, spleen colonization by *S. enterica* depended on the simultaneous exploitation of seven host nutrients - glycerol, fatty acids, N-acetylglucosamine, gluconate, glucose, lactate, and arginine (Steeb et al., 2013). During alfalfa seedling colonization, *S. enterica* was inferred to use glycerol, glucose, galactose, ribose, maltose, nucleosides, arginine, and proline to support its growth (this study), as mutants in these metabolic pathways were less fit. The exploitation of a greater range of sugars in the plant environment reflects the richness of sugars in plant exudates. In both plant and animal hosts, glycerol and its derivative glycerol-3-phosphate were metabolic drivers of *S. enterica* colonization. Mutants impaired in the transport and/or catabolism of these compounds were up to 10-fold less fit than the WT in competitive colonization of both hosts. Because these mutants were not non-competitive, we conclude that glycerol and glycerol-3-phosphate are not essential metabolic determinants of host colonization by *S. enterica*. Overall, most individual nutrients appeared to have small contributions to bacterial colonization. The availability or use of specific nutrients did not determine plant colonization ability; rather, high levels of *S. enterica* colonization is supported by the contributions of many plant-derived nutrients. This diversified metabolic strategy may enable *S. enterica* to colonize diverse hosts and better adapt to changes in the host environment, such as the alteration of plant exudate composition during plant development. However, the robust metabolism of *S. enterica* and the rich and dynamic nature of plant exudates present a complex challenge for designing strategies aimed at restricting or reducing enteric human pathogen growth on food crops. Identifying essential nutrients that *S. enterica* either cannot biosynthesize, e.g., minerals, or cannot import will be key to this endeavor.

While there are many similarities in *S. enterica* metabolism during plant and animal colonization, we highlight in this work three distinct differences in the metabolic networks used to support each bacterial lifestyle. For example, host differences and metabolic preferences lead to differential usage of the metabolic network surrounding fatty acids. Proteomic data indicate that this metabolic network is active during mouse spleen colonization and alfalfa seedling-associated growth (Steeb et al., 2013; Kwan et al., 2015). Fatty acid biosynthesis by *S. enterica* is likely important for repair of cell membranes damaged during infection and production of new membrane components for replicating cells. Disruption of fatty acid biosynthesis in a *fabB* mutant resulted in a 100,000-fold loss in competitive fitness (log_2_ (competitive index) < −16.7) and rapid clearance of the bacterium from infected mice (Becker et al., 2006; Barat et al., 2012). The failure of a *fabG* fatty acid biosynthetic mutant to grow during the first 24 h in alfalfa seedling exudates and the restoration of its growth ability thereafter suggests that fatty acids were not available to *S. enterica* in early seedling exudates but became available as the seedlings matured. Even as this nutrient became available, it was not a preferred nutrient source for *S. enterica* based on the WT phenotype of the *fadD fadK* fatty acid utilization mutant. This result is consistent with the use of sugars as preferred carbon sources, which are abundant in plant exudates. This conclusion contrasts with *S. enterica* nutrition during systemic murine infection where fatty acids were a major nutrient source (Steeb et al., 2013). Though fatty acid degradation was not necessary for the phases of plant-associated growth (lag to late log phases) examined in this study, partly due to the lack of this nutrient in very early seedling exudates, this metabolic function may become more important as bacterial cells enter the less biosynthetically-active stationary phase. At this point, maintenance and repair of cell membranes will likely shift from use of phospholipids synthesized *de novo* to modification or degradation and repurposing of existing cellular lipids. A recent study of stationary phase *S. enterica* in tomato fruits appears to support this conclusion (de Moraes et al., 2017). Fatty acid degradation may be an example of divergent use of a single metabolic network, for cellular maintenance vs. nutrition, during plant and animal colonization.

Serine metabolism is another example of a distinct difference between *S. enterica* plant and animal colonization. SerA and SerC, two enzymes involved in serine biosynthesis via the glycolysis intermediate 3-phosphoglycerate, are produced both during growth in germinating alfalfa exudates (Kwan et al., 2015) and during animal colonization (Becker et al., 2006; Steeb et al., 2013). In contrast, the serine transporter SdaC and serine deaminases SdaA and SdaB were detected only during animal infection. Mutant analysis indicates that serine biosynthesis is required for alfalfa root colonization (this study) but dispensable during murine infection (Jelsbak et al., 2014). Serine biosynthesis occurs during both plant and animal colonization but is only essential for root colonization suggesting that sufficient serine may be available in animals or serine synthesis from glycine is energetically favorable during animal colonization. The latter is supported by the finding that the *gvc* operon, involved in this glycine-to-serine conversion, is active during animal infection (Eriksson et al., 2003). Taken together, the data suggests that serine is limited in both murine spleens and alfalfa root exudates but *S. enterica* uses different biosynthetic pathways to overcome that deficit during its plant- and animal-associated lifestyles.

We also examined the metabolic network surrounding mannose. A *manA* mutant was severely compromised in competitive colonization of both plants and animals. ManA, or phosphomannose isomerase (Pmi), catalyzes the reversible isomerization of mannose-6-phosphate into fructose-6-phosphate, a glycolytic intermediate. In both plants and animals, the reaction catalyzed by ManA is believed to operate in the reverse direction, diverting fructose-6-phosphate from glycolysis to yield mannose-6-phosphate (Steeb et al., 2013, this study). Mannose-6-phosphate is a precursor for the nucleotide sugars that form the backbones of the common enterobacterial extracellular polysaccharides colanic acid and O-antigen. A *pmi* mutant is avirulent in mice, and the loss of competitive colonization fitness in a *manA* mutant is attributed to loss of O-antigen (Collins et al., 1991; Steeb et al., 2013). O-antigen delays immune recognition and confers resistance to complement-mediated killing and antimicrobial peptides in animal models of infection (Duerr et al., 2009; Ilg et al., 2009). However, we found no role for *manA* in resistance to ROS, acidity, flavone, or the antimicrobial peptide polymyxin B in plant exudates (data not shown). In fact, O-antigen deficiency only partially explained the phenotype of the *manA* mutant *in planta*. O-antigen contributed to seedling but not seed attachment, and competitive growth fitness in the plant environment but not the inherent ability to grow in the absence of competition. We also found no role for colanic acid in *S. enterica* seed or seedling attachment, colonization, or plant-associated growth. These results are consistent with a previous report that identified a role for O-antigen, but not colanic acid, in alfalfa sprout attachment and colonization by *S. enterica* sv. Enteriditis (Barak et al., 2007). However, colanic acid has been reported to contribute to desiccation tolerance on leaf surfaces (Cowles et al., 2016). The results from this study suggest that physiological functions in addition to O-antigen and colanic acid are impaired in a *manA* mutant, which is also reduced in seed attachment and severely compromised in the ability to grow during the first 24 h following inoculation into a germinating seedling culture. We hypothesize that mannose-6-phosphate and/or a downstream metabolite essential for plant-associated growth is absent from early seed exudates and must be biosynthesized endogenously by *S. enterica* to permit bacterial proliferation.

Taken together, this study shows that there is high overlap in the metabolic networks that are required for both the plant- and animal-associated lifestyles of *S. enterica*. However, some of these networks can be used differently to enable *S. enterica* to colonize each host, reflecting the chemical environment provided by the host. Common, simple molecules such as sugars, organic acids, and amino acids comprise the diet of *S. enterica* in both plants and animals. This finding was initially surprising to us because plants and animals are physiologically distinct and we expected unique *S. enterica* metabolic profiles in these environments. However, plants and animals share the same basic cellular metabolites (i.e., glucose, glycerol, amino acids, etc.) and pathways; it is understandable that similar host nutrients support *S. enterica* colonization of plants and the SCV inside of animal cells. Thus, the success of *S. enterica* in both host environments is partly due to similar available metabolites from plant and animal hosts. Additionally, the metabolic capacity of the bacterium—characterized by nutritional versatility, biosynthesis of limiting metabolites, and co-opting virulence pathways (e.g., the mannose metabolic network) for plant colonization—likely contribute to the ability of *S. enterica* to exploit cross-kingdom hosts.

## Conclusions

We have provided fundamental information about how a human enteric pathogen grows on and colonizes plants. This advancement is an important step to understand the biology of *S. enterica*, the leading bacterial pathogen of food-borne illness which is now most commonly linked to consumption of fresh produce. We examined *S. enterica* mutants defective in the metabolism of diverse nutrients for their ability to colonize plants in order to identify the traits that contribute to its success. A comparison with the metabolic requirements for splenic colonization revealed few differences in metabolic network use, e.g., amino acid and fatty acid metabolism and exopolysaccharide production. However, pathways within these networks served different functions facilitating S. *enterica* colonization of diverse niches. To thrive in distinct niches that span multiple kingdoms of life, *S. enterica* exhibits metabolic versatility and adaptation of physiological pathways for alternate functions. Having the capacity to colonize the food source (plants) of a preferred host (omnivore or herbivore) allows fluidity in the lifecycle of *S. enterica*.

## Author contributions

GK and JB conceived the study and wrote the manuscript. GK, JB, KC, TP, and DA-N analyzed the data. GK conducted the majority of the assays. TP conducted the metabolomics assays. BP and KC participated in mutant phenotyping. All authors have read and approved the final manuscript.

### Conflict of interest statement

The authors declare that the research was conducted in the absence of any commercial or financial relationships that could be construed as a potential conflict of interest.
