# Supplementary material for: Few Differences in Metabolic Network Use Found Between Salmonella enterica Colonization of Plants and Typhoidal Mice
Source: Front Microbiol. 2018 May 8;9:695. doi: 10.3389/fmicb.2018.00695 (PMC5951976; doi:10.3389/fmicb.2018.00695)
Supplement: Supplementary file 1 [file Presentation_1.PDF]

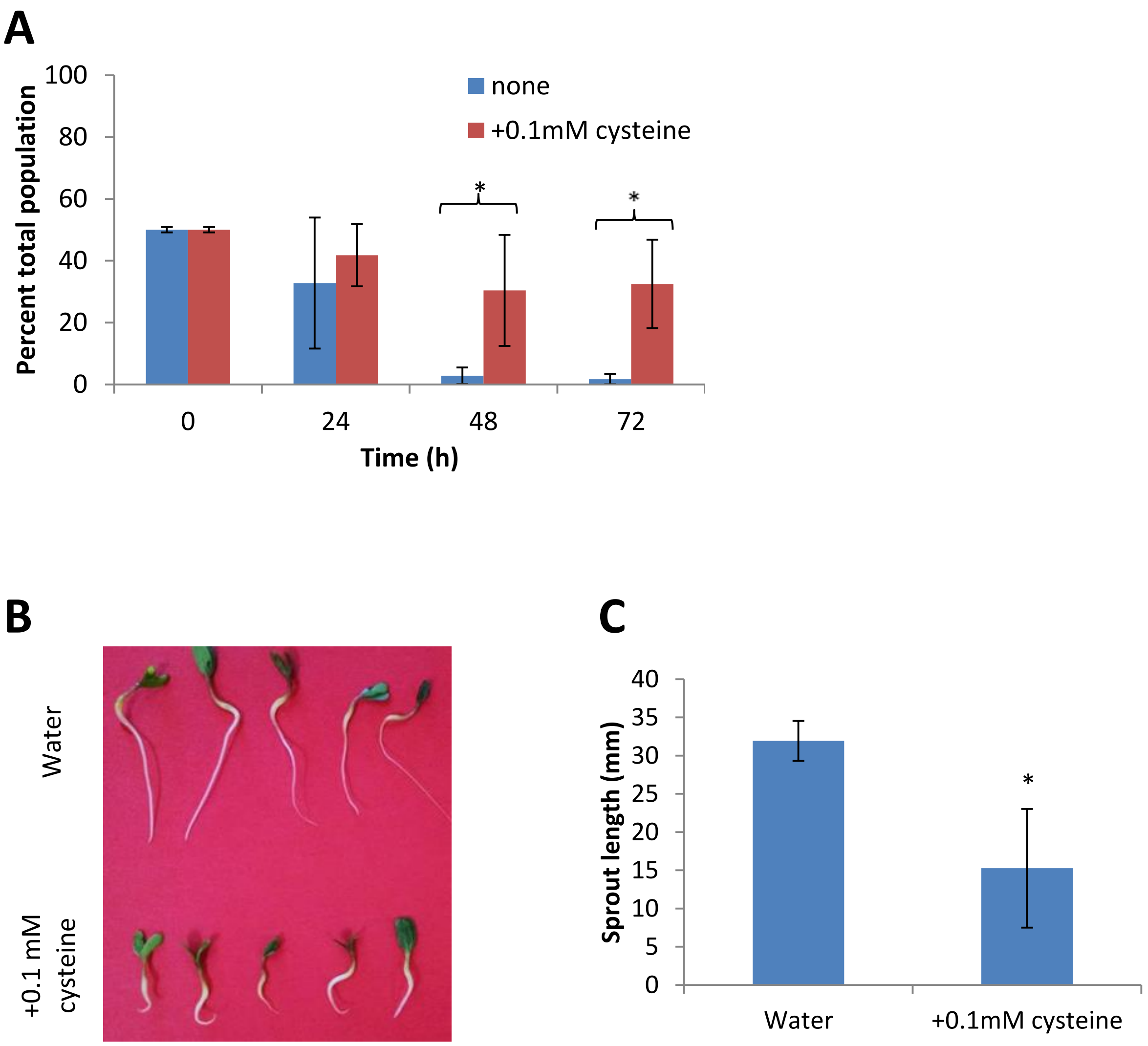


**Figure S1. Cysteine is toxic to alfalfa seedlings.** While the seedling colonization defect of a *cysE metA* mutant was partially complemented by addition of 0.1mM cysteine (A), the presence of this free amino acid caused significant stunting of alfalfa seedlings (B-C). Bars show the means of 3 independent experiments with 5 replicates each. Error bars indicate the standard deviation. *, difference is statistically significant (paired t-test, n=6, $p<0.05$).
