# Supplementary material for: Few Differences in Metabolic Network Use Found Between Salmonella enterica Colonization of Plants and Typhoidal Mice
Source: Front Microbiol. 2018 May 8;9:695. doi: 10.3389/fmicb.2018.00695 (PMC5951976; doi:10.3389/fmicb.2018.00695)
Supplement: Supplementary file 2 [file Table_1.DOCX]

**Table S1. Relative growth of amino acid auxotrophs compared with WT in lettuce and broccoli seedling exudates**

|  | **Relative growth of auxotroph (log_10_ CFU/ml)^b^** | | | | | | | | | |
| --- | --- | --- | --- | --- | --- | --- | --- | --- | --- | --- |
|  | **Lettuce** | | | | | **Broccoli** | | | | |
| **Mutant^a^** | **24 h** | **48 h** | **72 h** | **n** | **24 h** | | **48 h** | **72 h** | **n** |  |
| Δ*asnA*::Kan Δ*asnB*::Cm | -0.18±0.17 | -0.16±0.12 | -0.18±0.16 | 4 | -0.63±0.23 | | -0.38±0.22 | -0.33±0.06 | 4 |  |
| Δ*glyA*::*Tn*10d | -0.34±0.20 | -0.17±0.07 | -0.33±0.08 | 4 | -0.46±0.27 | | -0.48±0.23 | -0.22±0.01 | 4 |  |
| Δ*lysA*::Kan | WT | -0.26±0.11 | -0.56±0.16 | 4 | -0.72±0.08 | | -0.83±0.32 | -0.43±0.04 | 4 |  |
| Δ*metC*::Kan | -0.37±0.33 | -0.61±0.06 | -0.53±0.12 | 4 | -0.55±0.44 | | -0.66±0.30 | -0.38±0.04 | 4 |  |
| Δ*pheA*::Kan | -0.68±0.05 | -0.40±0.12 | -0.49±0.24 | 5 | -0.66±0.30 | | -0.48±0.14 | -0.20±0.10 | 4 |  |
| *proC693*::*MudA* | -0.15±0.05 | WT | WT | 4 | -0.9±0.08 | | -1.44±1.37 | -0.19±0.10 | 4 |  |
| Δ*trpB*::Kan | -0.33±0.21 | -0.23±0.06 | -0.13±0.18 | 4 | -0.76±0.08 | | -0.48±0.32 | -0.42±0.02 | 4 |  |
| Δ*tyrA*::Kan | -1.73±0.12 | -1.73±0.11 | -0.31±0.06 | 4 | -1.05±0.28 | | -0.72±0.05 | -0.43±0.05 | 3 |  |

^a^ All mutants are in the *S. enterica* 14028S background.
^b^ Values shown are the means ± SD. Population differences between mutants and the WT that are not statistically significant are shown as WT (paired t-test, n=6-10,p<0.05).
