# Supplementary material for: Few Differences in Metabolic Network Use Found Between Salmonella enterica Colonization of Plants and Typhoidal Mice
Source: Front Microbiol. 2018 May 8;9:695. doi: 10.3389/fmicb.2018.00695 (PMC5951976; doi:10.3389/fmicb.2018.00695)
Supplement: Supplementary file 3 [file Table_2.DOCX]

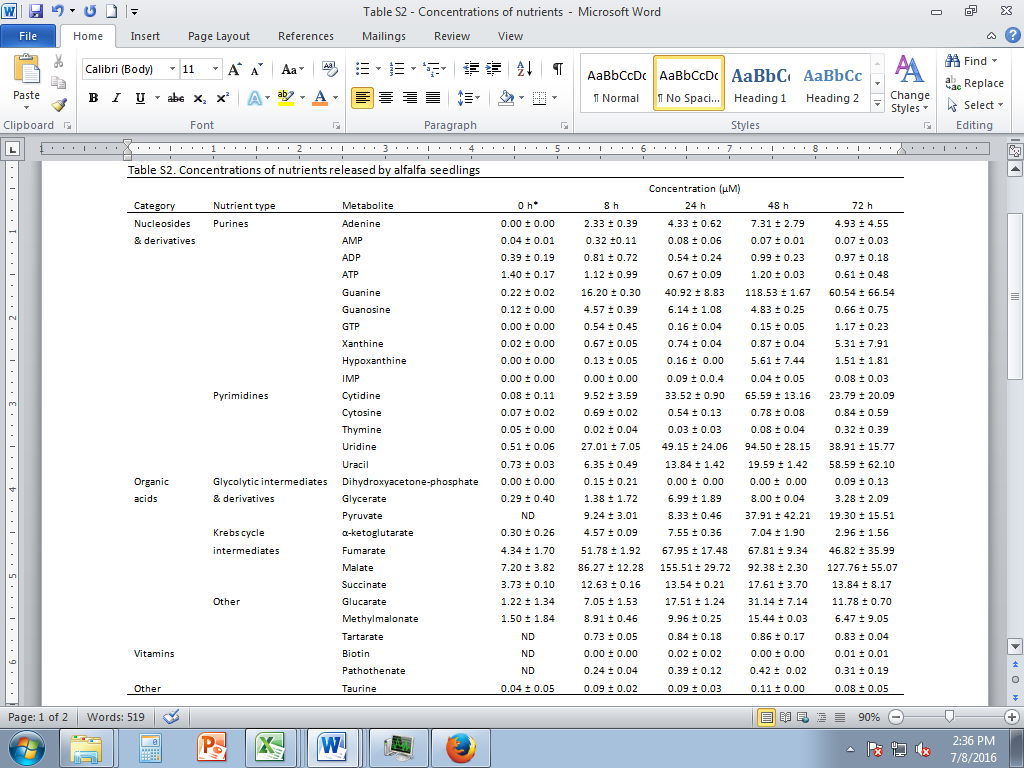


* Samples at 0h were collected immediately after addition of water to seeds. Nutrient concentrations at this time point likely reflect unbound nutrients present on the seed surface.

ND = metabolite not quantified at this time point
