# Supplementary material for: Few Differences in Metabolic Network Use Found Between Salmonella enterica Colonization of Plants and Typhoidal Mice
Source: Front Microbiol. 2018 May 8;9:695. doi: 10.3389/fmicb.2018.00695 (PMC5951976; doi:10.3389/fmicb.2018.00695)
Supplement: Supplementary file 4 [file Table_3.DOCX]

**Table S3. Relative depletion of nutrients in germinating alfalfa seedling exudates by *S. enterica***

| Nutrient type | Nutrient | Ratio of nutrient concentrations with Se : without Se (Avg ± SD) | | | | | | | | |
| --- | --- | --- | --- | --- | --- | --- | --- | --- | --- | --- |
|  |  | 24 h |  | 48 h |  | 72 h | |  | |  |
| Amino acid intermediates or derivatives | 2-isopropylmalic acid | 0.77 ± 0.28 |  | 38.85 ± 2.73 | * | 36.00 ± 30.52 | |  | |  |
|  | Aminoadipic acid | 0.96 ± 0.11 |  | 0.93 ± 0.04 | † | 1.03 ± 0.22 | |  | |  |
|  | Hydroxyphenylpyruvate | 1.03 ± 0.36 |  | 204.35 ± 77.83 | * | 79.87 ± 70.10 | |  | |  |
|  | N-acetyl-glutamate | 1.05 ± 0.14 |  | 4.11 ± 0.49 | * | 4.00 ± 0.90 | | * | |  |
|  | N-acetyl-glutamine | 1.14 ± 0.53 |  | 23.84 ± 4.30 | * | 19.55 ± 10.31 | | † | |  |
|  | N-acetlyl-L-alanine | 1.02 ± 0.05 |  | 1.22 ± 0.24 |  | 1.06 ± 0.16 | |  | |  |
|  | N-acetyl-L-ornithine | 1.00 ± 0.30 |  | 3.97 ± 0.61 | * | 4.03 ± 0.72 | | * | |  |
|  | Sarcosine | 1.02 ± 0.13 |  | 0.04 ± 0.01 | * | 0.04 ± 0.02 | | * | |  |
| Nucleosides and nitrogenous bases | Adenine | 0.62 ± 0.38 |  | 0.00 ± 0.00 | * | 0.00 ± 0.00 | | * | |  |
|  | Adenosine | 0.68 ± 0.28 |  | 0.00 ± 0.00 | * | 0.00 ± 0.00 | | * | |  |
|  | Cytidine | 0.90 ± 0.18 |  | 0.00 ± 0.00 | * | 0.00 ± 0.00 | | * | |  |
|  | Guanine | 0.93 ± 0.08 |  | 0.58 ± 0.04 | * | 0.57 ± 0.23 | | † | |  |
|  | Guanosine | 0.74 ± 0.16 |  | 0.00 ± 0.00 | * | 0.01 ± 0.01 | | * | |  |
|  | Hypoxanthine | 33.87 ± 31.52 |  | 62.31 ± 20.97 | * | 36.78 ± 30.30 | |  | |  |
|  | Inosine | 19.55 ± 12.00 |  | 0.00 ± 0.00 | * | 0.00 ± 0.00 | | * | |  |
|  | Thymidine | 0.93 ± 0.13 |  | 0.00 ± 0.00 | * | 0.00 ± 0.00 | | * | |  |
|  | Thymine | 4.40 ± 4.32 |  | 64.51 ± 21.98 | * | 81.32 ± 71.29 |  | |  |  |
|  | Uracil | 20.25 ± 24.68 |  | 85.77 ± 13.11 | * | 36.26 ± 32.24 |  | |  |  |
|  | Uridine | 0.92 ± 0.15 |  | 0.00 ± 0.00 | * | 0.00 ± 0.00 | | * | |  |
|  | Xanthine | 0.80 ± 0.33 |  | 0.27 ± 0.04 | * | 0.87 ± 1.08 | |  | |  |
|  | Xanthosine | 0.89 ± 0.06 | * | 0.84 ± 0.14 |  | 0.50 ± 0.11 | | * | |  |
| Organic acids | 2-dehydro-D-gluconate | 0.89 ± 0.10 |  | 0.18 ± 0.03 | * | 0.12 ± 0.07 | | * | |  |
|  | Aconitate | 0.87 ± 0.08 |  | 1.50 ± 1.13 |  | 0.07 ± 0.0 | | * | |  |
|  | Allantoate | 0.87 ± 0.17 |  | 1.08 ± 0.11 |  | 0.87 ± 0.29 | |  | |  |
|  | Citramalic acid | 91 ± 0.05 |  | 5.34 ± 0.63 | * | 3.10 ± 1.46 | |  | |  |
|  | D-gluconate | 1.19 ± 0.12 |  | 0.25 ± 0.04 | * | 0.14 ± 0.06 | | * | |  |
|  | Fumarate | 1.09 ± 0.16 |  | 0.07 ± 0.03 | * | 0.07 ± 0.06 | | * | |  |
|  | Glycerate | 1.49 ± 0.63 |  | 0.47 ± 0.23 | * | 0.15 ± 0.01 | | * | |  |
|  | Isocitrate | 1.17 ± 0.32 |  | 0.06 ± 0.03 | * | 0.03 ± 0.01 | | * | |  |
|  | Malate | 1.06 ± 0.13 |  | 0.04 ± 0.02 | * | 0.02 ± 0.02 | | * | |  |
|  | Methylmalonic acid | 1.21 ± 0.35 |  | 0.41 ± 0.26 | † | 0.25 ± 0.01 | | * | |  |
|  | Oxaloacetate | 0.85 ± 0.29 |  | 0.97 ± 0.11 |  | 1.09 ± 0.13 | |  | |  |
|  | Phenyllactic acid | 0.84 ± 0.10 |  | 57.05 ± 18.37 | * | 166.83 ± 144.28 | |  | |  |
|  | Succinate | 1.21 ± 0.35 |  | 0.41 ± 0.26 | † | 0.25 ± 0.01 | | * | |  |
| Vitamins | Biotin | 0.97 ± 0.21 |  | 0.00 ± 0.00 | * | 0.00 ± 0.00 | | * | |  |
|  | Pantothenate | 0.89 ± 0.06 |  | 0.79 ± 0.03 | * | 0.75 ± 0.14 | | * | |  |
|  | p-aminobenzoate | 1.01 ± 0.20 |  | 1.20 ± 0.28 |  | 1.19 ± 0.17 | |  | |  |
|  | Pyridoxal | 0.93 ± 0.10 |  | 1.17 ± 0.11 |  | 2.40 ± 0.80 | | † | |  |
|  | Thiamine-phosphate | 1.44 ± 0.11 | * | 0.37 ± 0.34 | † | 0.56 ± 0.20 | | † | |  |
| Other | 2,3-dihydroxybenzoic acid | 0.69 ± 0.05 | * | 0.78 ± 0.25 |  | 0.83 ± 0.16 | |  | |  |
|  | Allantoin | 1.05 ± 0.21 |  | 1.53 ± 0.06 | * | 1.61 ± 0.11 | | * | |  |
|  | Anthranilate | 1.01 ± 0.20 |  | 1.20 ± 0.28 |  | 1.19 ± 0.17 | |  | |  |
|  | Betaine | 0.73 ± 0.22 |  | 0.00 ± 0.00 | * | 0.01 ± 0.00 | | * | |  |
|  | Pyrophosphate | 0.18 ± 0.41 |  | 0.99 ± 0.46 |  | 0.81 ± 0.49 | |  | |  |
|  | sn-glycerol-3-phosphate | 1.10 ± 0.16 |  | 0.30 ± 0.05 | * | 0.15 ± 0.00 | | * | |  |

* indicates p<0.05 (one-tailed t-test, µ=1, n=3).

† indicates 0.05<p<0.1 (one-tailed t-test, µ=1, n=3).
