# Supplementary material for: Few Differences in Metabolic Network Use Found Between Salmonella enterica Colonization of Plants and Typhoidal Mice
Source: Front Microbiol. 2018 May 8;9:695. doi: 10.3389/fmicb.2018.00695 (PMC5951976; doi:10.3389/fmicb.2018.00695)
Supplement: Supplementary file 5 [file Table_4.DOCX]

**Table S4. Evidence for *S. enterica* utilization of specific host nutrients**

| Nutrient | Utilization period | Evidence |
| --- | --- | --- |
| Aspartate | 24 h, 48 h, 72 h | Measured depletion of aspartate at 24, 48, and 72 h in the presence of *S. enterica* (Kwan et al., 2015) |
| Glutamine | 24 h, 48 h, 72 h | Measured depletion of glutamine at 48 and 72 h; reduced growth of *glnH* transport mutant at 24, 48, and 72 h; growth of *glnA* auxotroph at 24, 48, and 72 h (Kwan et al., 2015) |
| Glycine | 24 h, 48 h, 72 h | Growth of *glyA* auxotroph at 24, 48, and 72 h (Kwan et al., 2015) |
| Proline | 24 h, 48 h, 72 h | Measured depletion of proline at 24, 48, and 72 h in the presence of *S. enterica*; growth of *proC* auxotroph (Kwan et al., 2015) |
| Serine | 24 h, 48 h, 72 h | Measured depletion of serine at 24, 48, and 72 h in the presence of *S. enterica* (Kwan et al., 2015); growth of *serA* auxotroph (this work) |
| Alanine | 48 h, 72 h | Measured depletion of alanine at 48 and 72 h (Kwan et al., 2015) |
| Arginine | 48 h, 72 h | Measured depletion of arginine at 48 and 72 h (Kwan et al., 2015); reduced colonization fitness of *artP hisP* transport mutant at 48 and 72 h (this work) |
| Asparagine | 48 h, 72 h | Measured depletion of asparagine at 48 and 72 h (Kwan et al., 2015) |
| Adenosine | 48 h, 72 h | Measured depletion of adenosine at 48 and 72 h (this work) |
| Guanosine | 48 h, 72 h | Measured depletion of guanosine at 48 and 72 h (this work) |
| Uridine | 48 h, 72 h | Measured depletion of uridine at 48 and 72 h (this work) |
| Inosine | 48 h, 72 h | Measured depletion of inosine at 48 and 72 h (this work) |
| Fumarate | 48 h, 72 h | Measured depletion of fumarate at 48 and 72 h (this work) |
| Glycerate | 48 h, 72 h | Measured depletion of glycerate at 48 and 72 h (this work) |
| Malate | 48 h, 72 h | Measured depletion of malate at 48 and 72 h (this work) |
| Succinate | 48 h, 72 h | Measured depletion of succinate at 48 and 72 h (this work) |
| Glycerol-3-P | 24 h, 48 h | Reduced competitive colonization fitness of *glpFK gldA glpT ugpB* transport/catabolism mutant at 24, 48, and 72h; glycerol transport/catabolism only had a modest contribution to fitness at 72 h (this work) |
| Glycerol | 72 h | Reduced competitive colonization fitness of *glpFK* transport/catabolism mutant at 72 h (this work) |
| Maltose | 24 h, 48 h, 72 h | Reduced competitive colonization fitness of *malG* transport mutant (this work) |
| Ribose | 24 h, 48 h, 72 h | Reduced competitive colonization fitness of *rbsB* transport mutant (this work) |
| Glucose | 24 h | Reduced competitive colonization fitness of *ptsG manX mglB galP* transport mutant at 24 h; Though this mutant is defective in transport of multiple sugars, comparison with the defects of *mglB galP* and *manX* indicates the defect at 24 h was due to *ptsG* (this work) |
| Galactose | 48 h, 72 h | Reduced competitive colonization fitness of *mglB galP* transport mutant (this work) |
| Panthothenate | 48, 72 h | Measured depletion of panthothenate at 48 and 72 h (this work) |
| Biotin | 48, 72 h | Measured depletion of biotin at 48 and 72 h (this work) |
| Pyridoxal-P | 24 h, 48 h, 72 h | Colonization by *pdxA STM0163* auxotroph at 24, 48, and 72 h (this work) |
| Thiamine | 24 h, 48 h, 72 h | Measured depletion of thiamine-phosphate at 48 and 72 h; colonization by *yabJ thiI* auxotroph at 24, 48, and 72 h (this work) |
